# A Multi-Core Controller for an Embedded AI System Supporting Parallel Recognition

**DOI:** 10.3390/mi12080852

**Published:** 2021-07-21

**Authors:** Suyeon Jang, Hyun Woo Oh, Young Hyun Yoon, Dong Hyun Hwang, Won Sik Jeong, Seung Eun Lee

**Affiliations:** Department of Electronic Engineering, Seoul National University of Science and Technology, Seoul 01811, Korea; jangsuyeon@seoultech.ac.kr (S.J.); ohhyunwoo@seoultech.ac.kr (H.W.O.); yoonyounghyun@seoultech.ac.kr (Y.H.Y.); hwangdonghyun@seoultech.ac.kr (D.H.H.); jeongwonsik@seoultech.ac.kr (W.S.J.)

**Keywords:** parallel recognition, federated learning, AI processor, embedded system, controller, distributed learning, independent operation

## Abstract

Recent advances in artificial intelligence (AI) technology encourage the adoption of AI systems for various applications. In most deployments, AI-based computing systems adopt the architecture in which the central server processes most of the data. This characteristic makes the system use a high amount of network bandwidth and can cause security issues. In order to overcome these issues, a new AI model called federated learning was presented. Federated learning adopts an architecture in which the clients take care of data training and transmit only the trained result to the central server. As the data training from the client abstracts and reduces the original data, the system operates with reduced network resources and reinforced data security. A system with federated learning supports a variety of client systems. To build an AI system with resource-limited client systems, composing the client system with multiple embedded AI processors is valid. For realizing the system with this architecture, introducing a controller to arbitrate and utilize the AI processors becomes a stringent requirement. In this paper, we propose an embedded AI system for federated learning that can be composed flexibly with the AI core depending on the application. In order to realize the proposed system, we designed a controller for multiple AI cores and implemented it on a field-programmable gate array (FPGA). The operation of the designed controller was verified through image and speech applications, and the performance was verified through a simulator.

## 1. Introduction

Recent advances in computer and semiconductor process technology have developed artificial intelligence (AI) technology. Due to this trend, adopting AI systems for a variety of applications such as speech recognition [1], medicine [2], and education [3] is widely expanding. Processing large-scale data is one of the basic features of AI technology. As using data centers to process these large-scale data is a widespread technique in AI systems, the demand for data centers is growing because of the increasing expansion of AI systems. Since the amount that the data centers are used is directly tied to the scale of the data, the necessity for systematic exploitation of the data, which dramatically helps to optimize the system to be smart, intelligent, and cost-effective [4], is emerging.

The general machine learning systems adopt the architecture in which the central server processes and trains most of the data and delivers the result to the client [5]. As the clients of this model rarely, if ever, perform the data preprocessing, the workloads of the system are concentrated on the central server. This characteristic makes the system use a high amount of network bandwidth due to sending a huge amount of data, which is rarely preprocessed, from client to server [6]. Moreover, the data communication between the clients and the server on these systems can cause security issues due to sending large amounts of data [7]. Hence, adopting this model to security-sensitive applications leads the system to use an enormous amount of additional system resources to grant security. In order to overcome these issues, a new AI model called federated learning was recently presented.

The term federated learning was introduced by McMahan et al. [8]: “We term our approach Federated Learning, since the learning task is solved by a loose federation of participating devices (which we refer to as clients) which are coordinated by a central server”. Unlike the previous model, federated learning adopts an architecture in which the clients take care of data training and transmit only the trained result to the central server. McMahan et al. [8] discuss an algorithm for federated learning, and Bonawitz et al. [9] discuss considerations for applying systems and algorithms for federated learning. The federated learning system repeats the training process which is typically driven by model engineers to develop a model for an application [10]. The general training process is as follows [10]:

1. Client selection: The server samples from a set of clients meeting eligibility requirements. For example, mobile phones might only check into the server if they are plugged in, on an unmetered Wi-Fi connection, and idle, in order to avoid impacting the user of the device.
2. Broadcast: The selected clients download the current model weights and a training program from the server.
3. Client computation: Each selected device locally computes an update to the model by executing the training program, which might, for example, run SGD on the local data (as in federated averaging).
4. Aggregation: The server collects an aggregate of the device updates. For efficiency, stragglers might be dropped at this point once a sufficient number of devices have reported results. This stage is also the integration point for many other techniques that will be discussed later, possibly including: a secure aggregation for added privacy, lossy compression of aggregates for communication efficiency, and noise addition and update clipping for differential privacy.
5. Model update: The server locally updates the shared model based on the aggregated update computed from the clients that participated in the current round.

The data training from the client, i.e., client computation, has the effect of encrypting the original data and reducing the amount of data [11,12]. This property makes it possible for the system to operate with reduced network resources [8,13] and reinforced data security [14,15,16]. According to these characteristics, federated learning is being applied to a variety of applications such as smart factories [17], edge device applications [18,19], and end user privacy-sensitive applications.

**Figure 1:**
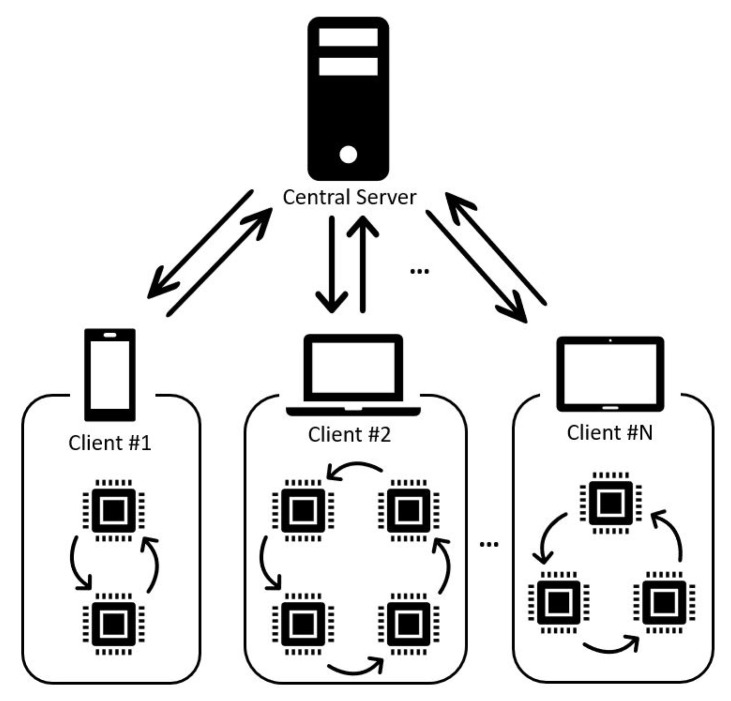
Concept of embedded AI system supporting federated learning.

Figure 1 shows the concept of an AI system with federated learning. Federated learning supports a variety of client systems and is able to perform client computation. Some of these client systems have resource limitations because of various factors. In order to execute client computation with these resource-limited client systems, dealing with these limitations is necessary. One of the applicable methods to solve these limitations is that of composing the client system with multiple embedded AI cores. Although federated learning between heterogeneous devices is the ultimate goal, parallel processing for federated learning among processors within a single device is prioritized, and the flexibility of the system can be provided by adopting this architecture. For the purpose of realizing the system with this architecture, the embedded AI cores need to be utilized and arbitrated by applying an additional controller [20]. In this paper, we propose an embedded AI system for federated learning that can be composed flexibly with AI cores depending on the application. The proposed system requires a specific module to process multiple AI cores in parallel. In order to verify the proposed system, we design a controller for a processor with multiple AI cores. 

The designed controller manages the signals for communication with commercialized AI chips and drives the states according to the learning/recognition commands. We validated the feasibility of the proposed system by implementing the controller on a field-programmable gate array (FPGA) and analyzed the performance of the controller by two applications, image recognition and speech recognition. Furthermore, we confirmed the validity of the controller and suitability of the application to the system through performance analysis according to the memory size using a simulator.

The main contribution of this work is as follows:

- Designing and verifying the controller for parallel processing of multiple AI cores.
- Constructing the protocol to transfer the training data and recognition data between the controller and the AI core.
- Performance analysis based on memory usage of the AI system with the *k*-NN algorithm.

As gathering recognition results from AI cores through the proposed controller, it helps to deduce the proper recognition result for the embedded AI system.

The paper consists of the following. Section 2 introduces related works about the AI system and simulator. Section 3 then turns to a detailed description about the AI algorithm and AI system architecture. Section 4 presents the implementation results of the system and analyzes the performance with the results. Section 5 compiles our entire work and draws conclusions.

## 2. Related Work

The recent research related to federated learning is categorized as follows:

- Artificial Intelligence for Embedded Systems;
- Simulating and Performance Analysis for Hardware Implementation.

### 2.1. Artificial Intelligence for Embedded Systems

Due to the limitations of embedded systems, much research in adopting AI in embedded systems concentrates on power and area optimization [21,22,23,24]. The research of [21] is one of the cases. The authors of this paper proposed an AI processor for embedded systems that was based on the k-nearest neighbor algorithm and operated by the coupled architecture with a master processor. By adopting the *k*-nearest neighbor algorithm, which has relatively small computations compared to other algorithms, the implementation of the AI processor was accomplished with a small area. The AI processor consists of memory cells which store vectorized raw data and distance calculators for classification. The performance and hardware specifications of the AI processor vary with the vector size of the memory cell and the total count of the memory cells. Hence, a framework to reconfigure these properties was built to provide the volatility of the hardware specifications. These properties, such as reconfigurable architecture and coupled architecture, make the AI processor applicable for a variety of applications. The research in [23] presents a dynamic reconfigurable processor (DRP) for embedded AI. The AI processor is composed of an array of processing elements (PE), multiply and accumulate unit (MAC) groups, and direct memory access (DMA). Each MAC group can be equivalent to small tensors or one large tensor in the deep neural network. The MAC supports the half-precision floating-point (FP16) and binary arithmetic mode. Selecting the binary arithmetic mode reduces memory usage but also degrades recognition performance. This architecture provides the flexibility of memory resource usage. In order to verify the DRP, the authors of [23] designed the system with the DRP, ARM CPU, and AXI interface and implemented it on a chip using 28 nm technology. The evaluation result of the chip shows that the DRP has acceptable performance on arithmetic in deep neural networks.

Unlike the research of [21,23], some studies focus on optimizing the software deep learning algorithm to fit existing embedded system-on-chip (SoC) [25,26,27]. Adopting the deep learning algorithm with applicable performance to the embedded SoC is extremely hard because of the critically limited memory and storage resources compared to cloud AI or mobile AI devices [25,26,27]. In order to fix these problems, the authors of [25,27] proposed frameworks for optimized neural network generation. Both frameworks provide quantization of floating-point arithmetic to integer arithmetic and applying memory constants for scaling the neural network for each device. The framework of [28] has two fundamental features. One is a neural architecture search (NAS). By providing an NAS that works with a memory size constraint, the neural architecture which is suitable to the microcontroller can be searched [25]. The other is a memory-efficient inference library. In contrast to traditional inference libraries, which depend on a runtime interpreter, the library adopts pre-runtime compilation to operate the system. This characteristic leads to significantly reducing the memory and computing overhead caused by the management of the metadata of variables. As a result, adopting the framework results in a remarkable reduction in latency and maximum SRAM usage. The framework in [27] concentrates on software optimization, which can be achieved by reflecting the configuration of mirror layered memory architecture and DMA provided by the target hardware. The result shows that the memory transferring overhead caused by cache memory is almost hidden. These studies suggest the possibilities of applying AI to the embedded SoCs.

### 2.2. Simulating and Performance Analysis for Hardware Implementation

When applying either AI or other features in embedded systems, searching and selecting hardware specifications that meet the performance requirements of each application are as important as designing the embedded system. Hence, many studies have been performed to build a framework to simulate and design the system efficiently [21,28,29,30]. The research of [29] is a suitable example of this research category. This paper presents a simulator to analyze the performance of the embedded AI to help to decide on the specifications of the embedded AI, such as the AI processor described in Section 2.1. The simulator can be executed with the sample dataset which is used for certain applications to secure the reliability of the results. As the specifications of the AI processor are related to memory size and the number of categories, the simulator provides the configuration of these options. As analyzing the performance after finishing the implementation of hardware is a far more complex process than analyzing with simulations, using the simulator to decide the attributes of the embedded AI before implementing the hardware increases the efficiency of the design and verification process of the system.

Many studies have been conducted that can be applied to federated learning as explained above. The main contribution of this work is as follows: to design and verify the controller of the embedded AI processors that is applicable to federated learning systems using the methodology of the above studies.

## 3. System Architecture

The embedded AI system performs learning and reasoning by using various datasets such as image or speech data. The embedded AI system allows the *AI core* selected by the user to operate AI functions (e.g., learning or recognition operations) through parallelized multiple *AI cores*. The *AI core* is a General Vision pattern recognition chip that performs recognition through the *k*-NN algorithm. In order to perform pattern recognition, the user should choose a method among the recognition stage and I2C slave controller and a method of directly controlling the neuron register.

In the proposed system, for the daisy-chain connection of several *AI cores* and optimization of the *controller*, the neuron register is directly accessed to control the neuro cell memory. For the purpose of utilizing the parallelized multiple *AI cores* efficiently, an *AI processor* needs a *controller* for multiple *AI cores*. Additionally, an *interface* is required to communicate data. The appropriate protocol for the *AI core* is used for the system to facilitate the repetitive AI operation. The algorithm and microarchitecture of the embedded AI system with operation flow are described below.

### 3.1. k-NN Algorithm

**Figure 2:**
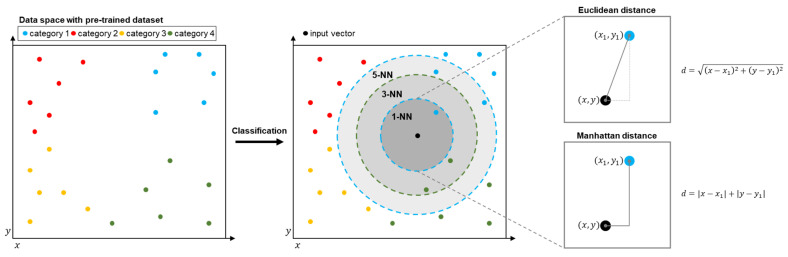
Visualization of the k-nearest neighbor algorithm.

The *k*-NN algorithm is a distance-based machine learning algorithm used for classifying the category of the data. The basic concept of the *k*-NN algorithm is that of finding the closest values between the input data and the trained dataset. The constant k in *k*-NN indicates how many values the algorithm will find. A distance calculation method between the trained data and input vector varies. In general, Euclidean distance or Manhattan distance is used for distance calculation. To classify the data with maintaining the concept of the *k*-NN algorithm, the information of the trained data and the input data, which contains a variety of characteristics, needs to be expressed to a group of numerical values and a single category of information. Thus, the data which is used for the algorithm is expressed as a group of n-dimensional vectors and the category. Figure 2 presents the mechanism of the *k*-NN algorithm when the data are expressed as a two-dimensional vector. The algorithm operates by two key features. One is training the system with the algorithm by storing the vector data with category information. The other is a classification of the input data. 

Algorithm 1 presents the classification process. During the classification, the *k*-NN algorithm finds a *k* number of data with the shortest distance in the trained dataset. After finding the nearest data, the algorithm extracts the most frequent category from a number of nearest data. Due to the architecture of these key features, the most frequent category can be more than one when the *k* value is more than one. To avoid this problem, an additional method to give proper weight to the found data or setting the *k* value to one is required. As shown in Algorithm 1, an increase in the constant *k* leads the complexity and iteration count of the algorithm to be higher. This attribute is not appropriate for the embedded system which is sensitive to area usage and power consumption. Therefore, we set the *k* value to one to minimize the area usage and power consumption of the system. Algorithm 2 shows the minimized algorithm by setting the constant *k* to one.

**Algorithm 1:***k*-NN classification algorithm pseudo-code

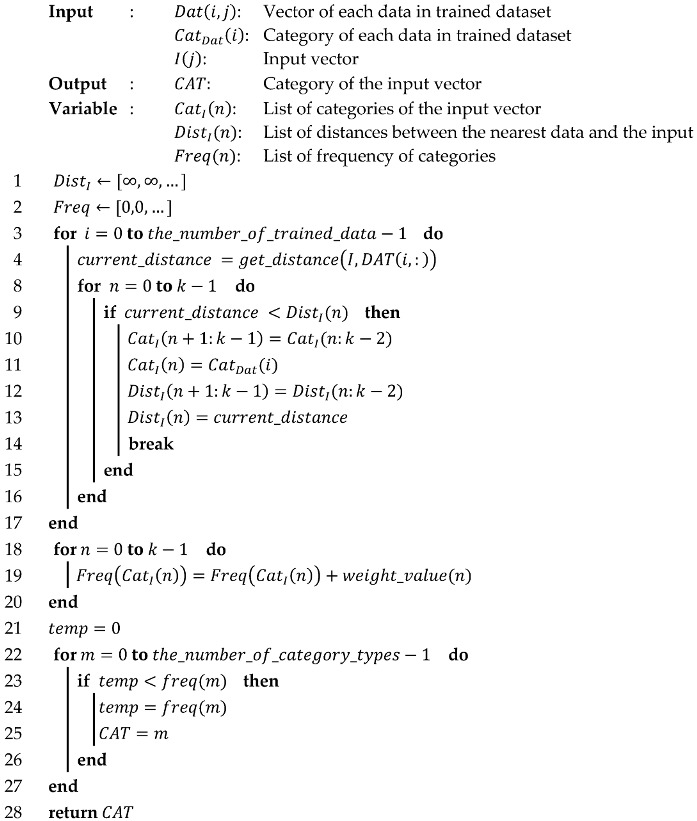



**Algorithm 2:***1*-NN classification algorithm pseudo-code

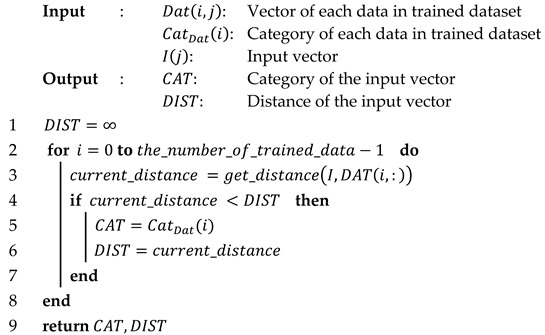



The *AI core* performs key features of the *k*-NN algorithm except extracting from the most frequent category. It provides the category and distance values sequentially in order of the shortest distance between the input vector and the data from the trained dataset. The specifications, such as the constant *k* and number of dimensions, can be configured by an external device. Additionally, the *AI core* applied the Manhattan distance method to lower the complexity of calculation in order to operate with low power consumption. The *AI core* can have 1024-byte vectors that are composed of up to 256 components of 8-bit value, and vectors can include 15-bit of category information [31]. Each datum in the vector is expressed as a 1-byte unsigned integer.

### 3.2. Microarchitecture

**Figure 3:**
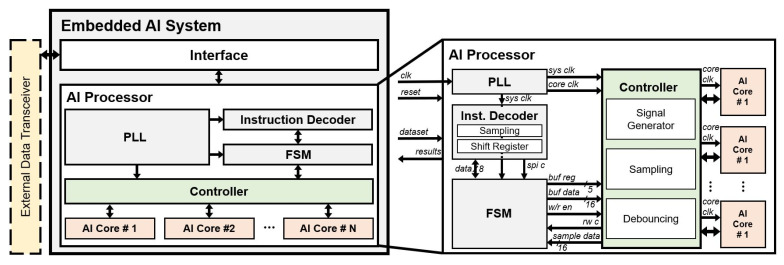
Block diagram of embedded AI system and *AI processor* in detail.

Figure 3 illustrates the block diagram of the embedded AI system. The embedded AI system includes an *interface* for communication with other systems through an external data transceiver and an *AI processor* with multiple *AI cores* for learning/recognition operations.

The external data transceiver sends a certain dataset for learning and recognizing operations, which is shaped to a protocol based on a serial peripheral interface (SPI) protocol, to the *interface*. The AI processor includes the *phase locked loop* (*PLL*), *instruction decoder*, *finite state machine* (*FSM*), *controller* for efficient management of *AI cores*, and *AI cores*. The *PLL* generates a 64MHz system clock for the *controller* and a 16MHz core clock for the *AI cores*. The *instruction decoder* in the AI processor receives the dataset through the *interface* by decoding a protocol for learning/recognition.

**Figure 4:**
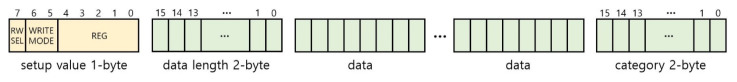
Protocol for learning and recognition in detail.

Figure 4 shows the protocol for learning and recognition. The protocol basically contains a 1-byte setup value composed of configurations such as read/write select (*RW SEL*), *write mode*, and register address (*REG*). *RW SEL* is required to distinguish between learning and recognition operations by the *FSM*. After the division operation, the system decides whether it is single-mode or not using *write mode*. In the case of learning in single-mode, the setup value to configure the *interface* to single write mode and set the destination to a certain address is first sent to the *interface*. Next, two bytes of the learning data are sent. In the case of learning in sequential mode, the setup value to configure the *interface* to sequential write mode and set the destination to a certain address is first sent to the *interface*. After sending the setup value, the 2-byte length value of learning data and a number of learning data are sent sequentially. When an *AI core* operates learning or recognition, a 5-bit register address, data, and category value are transmitted via control lines [31] through the *FSM* and the *controller*. Due to the protocol, the *FSM* and the *controller* generate control signals and send data for *AI cores* without storing the dataset.

**Figure 5:**
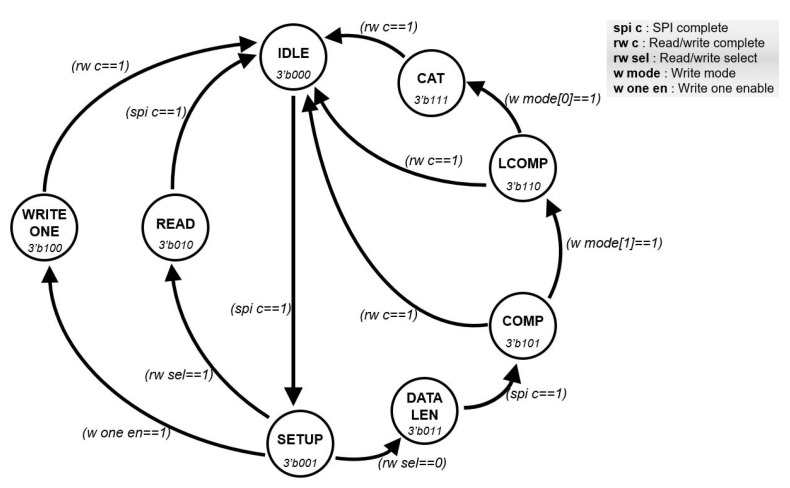
The architecture of the FSM.

The *FSM* is a module to provide well-ordered communication between the *instruction decoder* and the *controller*. Figure 5 shows the *FSM* in detail, which operates according to the active signal from the *instruction decoder*. The *FSM* performs learning and recognition operations based on the serial interface and distinguishes between learning and recognition operations. Furthermore, the *FSM* can discriminate between single and sequential modes. In case of the single-mode, the *FSM* only needs 2-byte data to receive read data or transmit write data. In the other case, the *FSM* requires 1-byte data for data length and continuous data including category data. When the result data from the *AI core* are received through the *controller*, the result data are sent to the *instruction decoder*. In order to perform the learning/recognition process or communication when the *FSM* transmits data to the *controller* or *instruction decoder*, it receives a flag signal from each module to prevent data collision. In addition, the *FSM* generates different signals according to the learning/recognition process so that the user can control the neuron register of the *AI core*. The architecture of the *FSM* allows the AI processor to perform continuous learning and recognition operations.

The *controller* is connected in parallel with the multiple *AI cores*, and each *AI core* can be operated independently through the *controller*. The *controller* generates control signals that can directly control the operation of the *AI core* [31] and transmits it along with the data. In the case of the learning process, the *controller* transmits the data to the selected *AI core* when it receives the *buffer data* and the *write enable* signal from the *FSM*. In the case of the recognition process, the *controller* transmits the data and *read enable* signal to the selected *AI core*. After that, the *controller* receives a result of the distance calculation and the category value from the selected *AI core* and transmits them to the *FSM*. When the *controller* receives the result data from the *AI cores*, the *sampling* block is performed to receive data reliably.

The *AI core* needs to receive a dataset (*vector*) before performing learning or recognition. The method of transmitting the vector to the *AI core* is called vector broadcasting [31]. In vector broadcasting, the processes called the component (COMP), which stores 1-byte data, and the last component (LCOMP), which is performed after COMP is finished, exist. The COMP can be performed up to 255 times. In the case of learning, the state called the CAT, which stores the category data, is performed after the vector broadcasting is finished. After the learning operation, the *AI core* that receives the test *vector* calculates the distance between learned and test data. The *AI core* reads the category value from the recognition result of the READ state, which has the lowest distance value.

## 4. Implementation and Analysis

**Figure 6:**
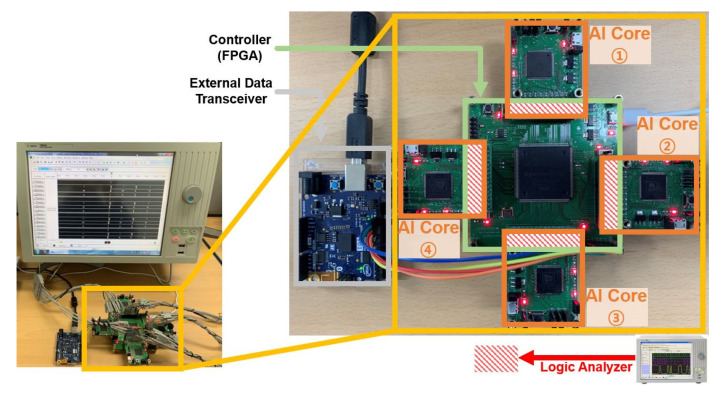
Experimental environment of the FPGA implementation.

In order to evaluate the embedded AI system, we implemented an *AI processor* which includes multiple *AI cores*. Figure 6 shows the hardware experiment environment in which the *controller* and multiple *AI cores* are connected in parallel. We implemented the *controller* on an Intel FPGA (Cyclone III) and used General Vision’s pattern recognition chip (CM1K) for the *AI core*. Using the verified *AI core* helps to clarify the functional verification of the designed *controller*. Previous to testing the implemented processor, we simulated the processor using a simulator written in Python that has the same capability as the *AI core* for performance analysis. Before applying the application to the embedded AI system, the simulator that mimics the structure of the *AI core* can be used to check the suitability of the application. We analyzed the performance according to the memory size of the virtual *AI core* and performed functional verification of the system through image and speech applications.

### 4.1. Implementation

#### 4.1.1. Case Study 1: Image Recognition

The MNIST dataset of handwritten digits has a training set of 60,000 examples and a test set of 10,000 examples. The digits are in a grayscale image, size-normalized, and centered in a fixed-size image [32]. In order to uniformly learn the *AI core* by category, data are evenly sampled from the MNIST dataset. The sampled training data are used for each *AI core* to learn.

**Figure 7:**
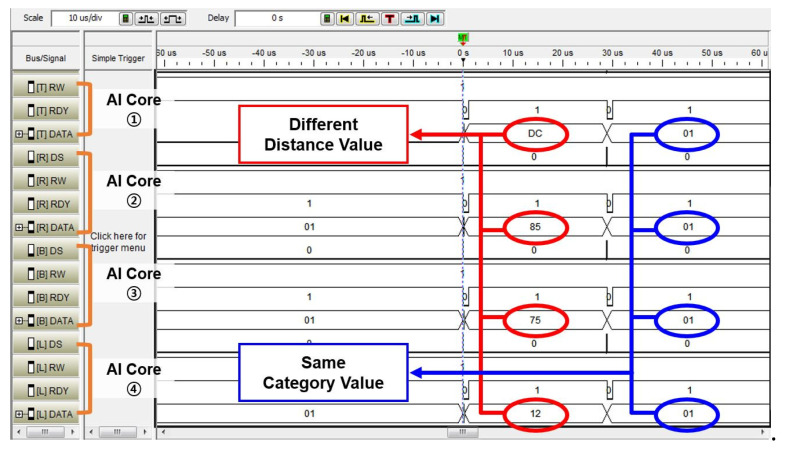
Image recognition results on FPGA implementation.

With the purpose of verifying the functionality of the designed *controller* in the image application and the parallel processing of multiple *AI cores*, we constructed the environment of the implementation. Figure 7 shows the result of measuring the signals through the logic analyzer for the distance result value and the category value of each *AI core*. The recognition results from each *AI core* are collected to the *controller*. Each *AI core* learned the same category (from 0 to 3) through different training data and received the same test data for recognition.

Each *AI core* outputted a result of distance calculation between the trained data and test data through the *k*-NN classifier in the *AI core* and a category value accordingly. As a result of the recognition, it was confirmed that the result values of the distances 0 × DC, 0 × 85, 0 × 75, and 0 × 12 are sequentially from the first *AI core*, and the category values of all *AI cores* are 0 × 1, indicating the same value. The distance calculation results from the *AI cores* were obtained by the *k*-NN algorithm in Section 3, and each *AI core* evaluates a category value based on the distance calculation result. This meant that the trained data of *AI core* (1) was furthest from the test data, and the trained data of *AI core* (4) was most similar to the test data. The *AI cores* were operated independently by the *controller* as each AI core outputted a difference distance value. As a result, the *controller* gathers the different distance values and same category values from each *AI core*.

#### 4.1.2. Case Study 2: Speech Recognition

As speech technology advances, research of speech recognition is increasing, but the availability of datasets has not widened [33]. In order to train and evaluate the embedded AI system for a speech application, the speech command built in Google Brain [33] is used as follows:

- Google Brain’s 2 categories Speech Command;
- Google Brain’s 3 categories Speech Command;
- Two categories of Recorded Voice Data.

In this experiment, the above speech commands were selected in consideration of the embedded AI system. Each speech command is evenly sampled to train the *AI core* by category. The sampled data is transformed into a two-dimensional (2D) signal through the short-time Fourier transform (STFT). The 2D signal is resized to be used as an input to the *AI core* and converted into a 1D signal. Finally, the converted data is used in the embedded AI system. In order to verify the functionality of the *controller* in the speech application, the experiment was implemented as in Case 1 (image application). Each *AI core* learned Case 2-3 (two categories of recorded voice data) training data recorded in different voices and recognized the test data. As the result, the *controller* for multiple *AI cores* operated successfully in the speech application.

### 4.2. Analysis

Table 1 shows the power consumption and logic element usage of the *controller* on the Intel EP3C25Q240C8N FPGA and the power consumption of the *AI core*, which is the General Vision chip [31]. The synthesis of the *controller* was executed with Quartus II 13.1 version software. The power consumption of the *controller* was obtained with PowerPlay Power Analyzer in Quartus II 13.1 version. The result presents that the proposed system is suitable for an embedded device.

**Figure 8:**
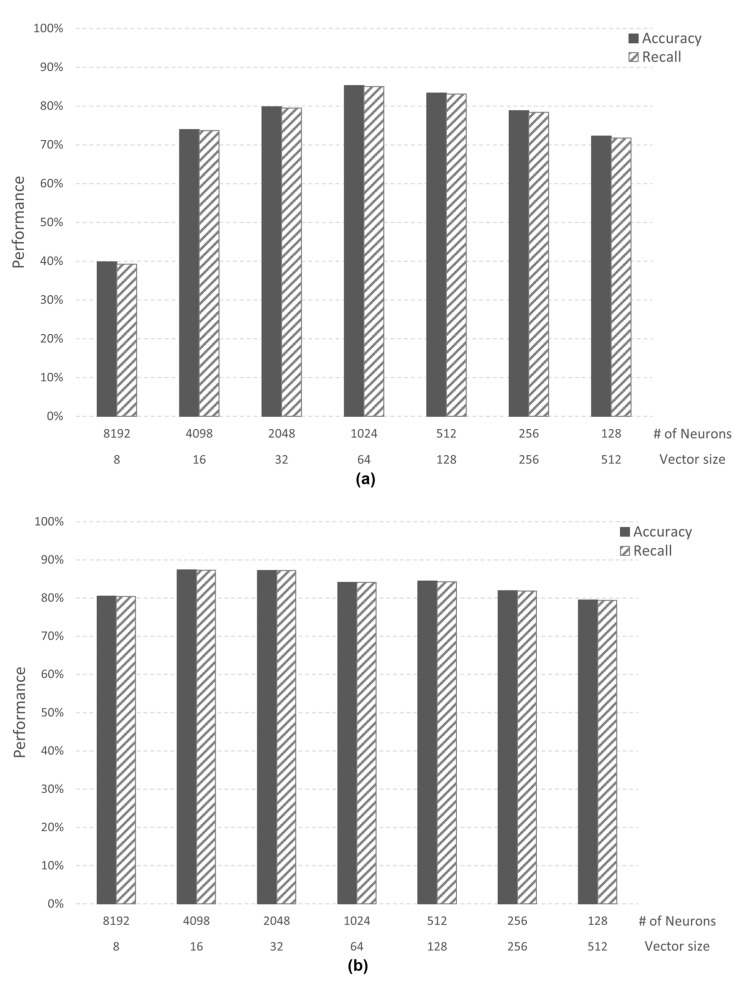
(**a**) Case 1 performance of recognition; (**b**) Case 2–1 performance of recognition.

The performance was verified by assuming a virtual AI core with a memory size of 16 KB and 64 KB through a simulator. The experiment was conducted for each case above, and learning was conducted using only the training data of each dataset. In order to evaluate the performance of each case, the value of accuracy and recall, one of the performance indicators of the AI algorithm, was measured. In addition, we used datasets for learning and evaluation for performance evaluation. Case 1 (image application) trained 10 categories of the MNIST dataset, and Case 2-1 (speech application) trained two categories of speech command data from Google Brain. Figure 8 shows the performance according to the data size (*vector size*) and the number of neurons (*# of neurons*) in the *AI core*. 

**Figure 9:**
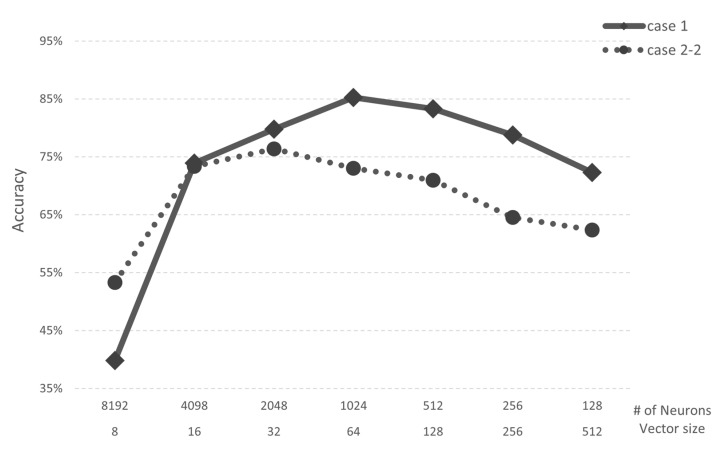
Accuracy of Case 1 and Case 2-2.

In Case 1, when the *vector size* is 64 bytes and the number of neurons is 1024, the *AI core* has both an accuracy and recall of 85%. In Case 2-1, when the *vector size* is 16 bytes and the number of neurons is 4098, the *AI core* has both an accuracy and recall of 87%. However, since the *AI core* experimented with has a maximum number of neurons of 1024, the maximum performance in Case 2-1 is 84%. Through this analysis, we confirmed that the minimum vector size required in both Cases is 64 bytes. Case 2-2 (speech application) trained three categories of speech command data from Google Brain. In Case 2-2, when the *vector size* is 64 bytes and the number of neurons is 1024, both accuracy and recall are 73%. Case 2-2 is confirmed to have lower performance than Case 2-1 in the same simulator environment because there are more categories than Case 2-1. Figure 9 shows the accuracy of Case 1 and Case 2-2. When the vector size is 8, Case 2-2 shows higher performance than Case 1. However, when the vector size is 16 or higher, Case 1 shows better performance than Case 2-2.

**Figure 10:**
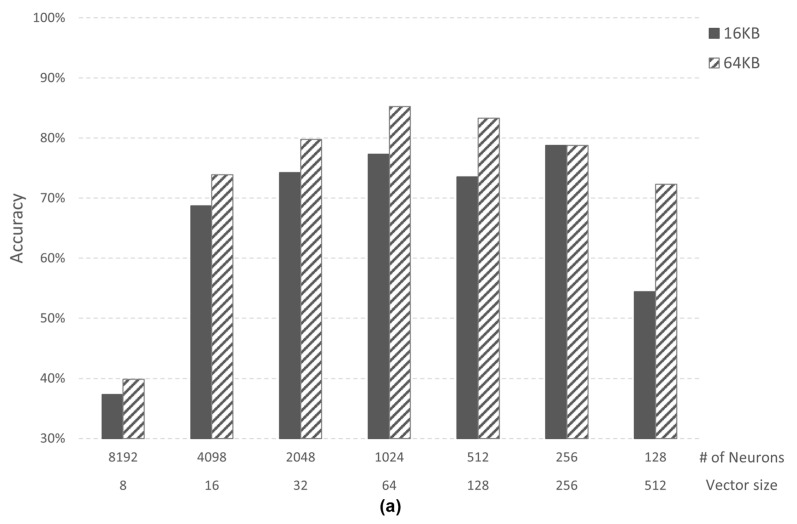

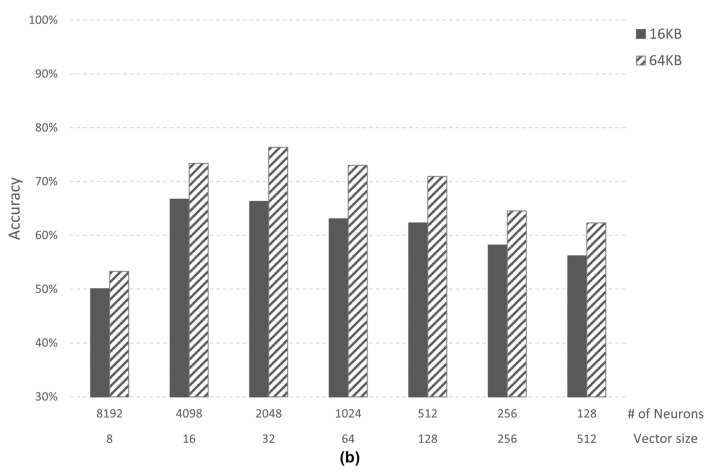
(**a**) Accuracy comparison in Case 1; (**b**) Accuracy comparison in Case 2-2.

Figure 10 shows accuracy analysis according to 16 KB and 64 KB memory size of the *AI core* in Case 1 and Case 2-2. The number of neurons (*# of neurons*) changes according to the memory size since the vector size is fixed. The accuracy of 64 KB memory shows higher accuracy than 16 KB memory in both cases. In Case 1, the accuracy of 64 KB memory is up to 17.87%p (percentage point) higher than 16 KB memory, and Case 2 is 10.09%p higher.

Through the experiment, the suitability of the application could be verified before being applied to the hardware, and the validity of the controller was verified through performance analysis according to the memory size. In addition, it was confirmed that the controller of the proposed system operates successfully, and the parallel processing functionality in the embedded AI system was determined.

**Figure 11:**
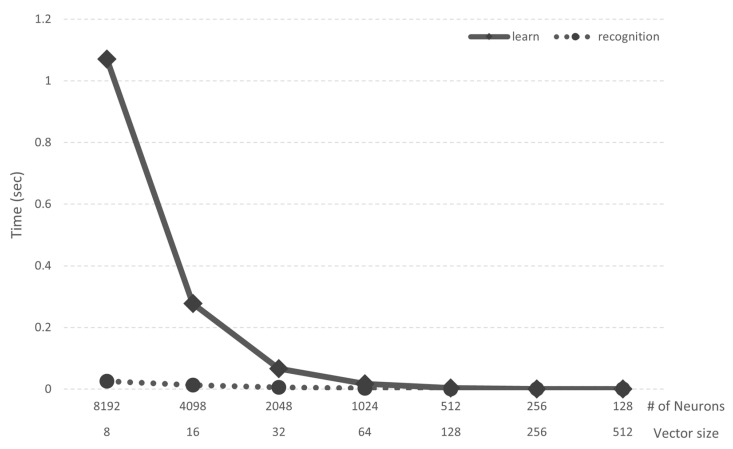
Time consumption of training and recognition.

Since we used the *k*-NN algorithm, the time it takes to learn and infer data, except preprocessing, is largely independent of the class of data. We measured the time taken for learning and inference using the MNIST dataset, and the results are shown in Figure 11. We used the simulator to measure the time, and the system specifications were an Intel i9-11900K (Intel Corporation, Santa Clara, CA, USA), 94.2 GB Memory, and an Nvidia RTX-3090 (Nvidia Corporation, Santa Clara, CA, USA). When the number of neurons is 8192, the training time is 1.071 s. As the vector size decreases, the time required for learning decreases dramatically: 0.278 s when there are 4098 neurons. Thus, the case of 1024 neurons shows the highest performance: it takes only 0.017 s for learning and 0.003 s for recognition. The recognition times show less than 0.03 s regardless of the number of neurons.

## 5. Conclusions

Federated learning enables distributed clients to learn prediction models while collecting training data on the clients. For this, an embedded system that supports federated learning is required. This paper proposed a controller for parallel recognition among AI cores within an embedded AI system. The AI processor in the embedded AI system contains several AI cores in parallel and a controller for efficient operation of the AI cores. The controller independently operated multiple AI cores and flexibly configured the number of AI cores according to the requirements of the application to be applied. In addition, the performance and limitations were determined in advance through virtual artificial intelligence cores and simulators. In order to verify the functionality of the designed controller, image and voice recognition were performed in the FPGA, and the performance according to the number of neurons and data size in the AI core was confirmed through the simulator. According to hardware design and the analysis, it is possible to confirm the potential of the embedded AI system in which multiple artificial intelligence cores can be united. Furthermore, the embedded AI system can choose the proper recognition result from gathered recognition results by the controller through polling or a comparison among collected results.

In future work, we will apply our controller for an embedded intelligence processor to various embedded devices and experiment with the association between heterogeneous embedded systems.

## Figures and Tables

**Table 1:** Resource utilization of the AI processor.

	Resource		Synthesis
Controller (Cyclone III: EP3C35Q240C8N)	Total logic elements ^1^		182 (<1%)
	Core dynamic power consumption		1.62 mW
	Core static power consumption		81.03 mW
AI Core (CM1K ^2^)	Single chip	Idle mode	15 mW
		Active mode	~275 mW
	Multiple chip	-	~500 mW

^1^ Total of 24,624; ^2^ referenced General Vision document.
